# Psychometric Validation of the Revised Physical Self-Perception Profile: An Italian Context Study †

**DOI:** 10.3390/bs14121229

**Published:** 2024-12-20

**Authors:** Simona Nicolosi, Rosario Ortega Ruiz, Francesco Sgrò, Mario Lipoma, Juan de Dios Benítez Sillero

**Affiliations:** 1 Department of Human and Social Sciences, Kore University of Enna, 94100 Enna, Italy; francesco.sgro@unikore.it (F.S.); mario.lipoma@unikore.it (M.L.); 2 Research Group in Sport Psychology for Well-Being and Health, 94100 Enna, Italy; ed1orrur@uco.es (R.O.R.); eo1besij@uco.es (J.d.D.B.S.); 3 Faculty of Education and Psychology, University of Cordoba, 14071 Cordoba, Spain; 4 Laboratory of Studies on Coexistence and Prevention of Violence (LAECOVI), 14071 Cordoba, Spain; 5 Investigation Group on Sport and Physical Education for Personal and Social Development (GIDESPO), 14071 Cordoba, Spain

**Keywords:** physical self-concept, perceived competence, perceived importance profile, reliability and validity, Italian students

## Abstract

Background: The Physical Self has been defined as the set of perceptual, descriptive, and evaluative aspects that each person uses to describe his or her physical domains. The Revised Physical Self-Perception Profile questionnaire (PSPP-R) is a self-reported measure of physical self-concept domains, including a Perceived Importance Profile questionnaire that assesses the subjective importance assigned to physical domains. Although the PSPP-R has been validated in several countries, it has not been validated in the Italian context. The present study aimed to test the psychometric properties of the Italian version of the PSPP-R with the PIP in order to provide, for the Italian context, a comprehensive instrument for understanding the development of the Physical Self and global self-esteem dimensions. Methods: The factor structure, internal consistency, temporal stability, and criterion validity of the Italian version of the PSPP-R were examined in two independent samples of Italian students (*n* = 911; 348 males and 563 females) aged 15–20 years attending secondary school. Results: The results demonstrated the validity and reliability of the Italian version of the PSPP-R, as well as its hierarchical structures. Conclusions: In psychological and educational settings, the Italian version of the PSPP-R can be used to examine the physical self-concept of Italian adolescents and young adults, allowing for the exploration of the interplay of competence and importance in physical domains.

## 1. Introduction

The Physical Self has been defined as the set of perceptual, descriptive, and evaluative aspects that each person uses to describe his or her own physical domains [1]. When the result of its evaluation is positive, the physical self-concept provides security, confidence, and a reasonable level of self-esteem. Although this construct is relatively stable, it is subject to developmental changes. During the course of development, the individual activates his or her self-reflective capacities through conducting an increasingly complex, generalized, and organized synthesis of his or her own bodily experiences. This depends on various facets (e.g., physical appearance characteristics, body weight, sports skills, state of health) and different contexts of action (e.g., private and public, competitive sport or recreational play). In the adolescent years, when psychosocial processes acquire important values for well-being and mental health, having a good approximation of self-concept is important.

Following Fox’s [2] definition, bodily self-description is the subjective reading of body characteristics and physical abilities expressed in experiences related to the body or any other aspect related to it. Physical self-concept can be considered the result of reworking the complex of these experiences.

During adolescence, the physical changes that occur during puberty, associated with individual and environmental factors, also involve a reworking of the bodily dimension of the Self, which has profound repercussions on the global Self, socio-relational styles, emotionality, the definition of choices, and behaviors. Adolescence is the only phase of life that involves massive bodily changes in an individual capable of making sense of and reflecting on his or her changes. Moreover, the changes linked to puberty are loaded with social and cultural meanings related to the development of an adult body. Somatic changes, in fact, acquire importance according to the responses given by the subject themself and those around them. The transition phase between puberty and the onset of adolescence, in particular, becomes a crucial phase for the implementation of training programs aimed at promoting psychophysical well-being [3,4,5].

Several authors have shown that the physical self-concept is linked to physical activity practice and a variety of physical fitness indicators [6,7,8,9,10]. Physical competence also has an impact on self-concept. Muscle development potentially increases physical strength, motor skills, and sports performance, which can have effects on perceived physical efficiency and also on the adolescent’s social relationships, with different results in relation to gender [11]. In children, the potential presence of barriers to physical activity participation is constituted by low actual and perceived physical competence, low levels of physical fitness, and obesity [9]. Several studies have also illustrated the nature of the relationship between physical activity and perceived and actual physical competence [12,13,14]. These results support the findings of the study by Stodden et al. [15], who suggested that perceived physical competence is a mediating variable in the relationship between actual physical competence and physical activity practice. According to the results of a meta-analysis by Babic et al. [16], in childhood and adolescence, physical activity is strongly associated with perceived competence, followed by perceived physical condition (fitness) and physical appearance. Physical appearance is the least related to physical activity. Consequently, actual motor skills may promote a positive relationship between perceived motor skills, physical activity, and physical fitness in childhood and adolescence [9,16,17]. These results suggest that, within this relationship, a positive or negative spiral of healthy behaviors in childhood and adolescence can be generated [14,16,17,18,19].

Empirical studies on the Physical Self have been carried out using self-reported instruments. In later decades, specific instruments have been constructed to measure physical self-concept, such as the Physical Self-Description Questionnaire (PSDQ) by Marsh [20], the Physical Self-Concept (PSC) by Richards [21], and the Physical Self-Perception Profile (PSPP) by Fox and Corbin [1], based on Harter’s [22] Self-Concept instruments. In these three measures, the physical self-concept construct is organized as a multi-dimensional and hierarchical structure. Marsh’s PSDQ has eleven scales (Self-Esteem, Physical Self-Concept, and nine Physical Self-Concept subscales, including health, physical appearance, body fat, physical activity, sports competence, coordination, flexibility, strength, and endurance). Richards’s PSC measures seven dimensions, including activity, appearance, health, competence, strength, body build, and satisfaction. In contrast, within the PSPP theoretical framework, global self-esteem is a superordinate domain above physical self-esteem. This latter comprises four specific subdomains: body attractiveness, sports competence, physical strength, and physical conditioning [1]. In a previous study, Marsh et al. [20] conducted a comparison of the three instruments using MTMM analyses to demonstrate their convergent and discriminant validity. In the same study, the PSPP was found to be the psychometrically weakest of the three measures among Australian high school students. The PSPP has been criticized [23] because of methodological issues arising from the idiosyncratic response scale used to measure responses to the items. This type of scale can introduce additional complexities in interpreting responses and may make it more difficult to compare with the results obtained using more traditional scales. In fact, participants are required to first choose between two statements and then decide the degree to which they agree with the chosen statement [24]. On the basis of the shortcomings of the previous PSPP, a revised version (PSPP-R) was developed by Hagger et al. [25]. It uses a traditional Likert-type scale instead of the problematic idiosyncratic response scale. Furthermore, only positively worded items are used in order to minimize method effects. These revisions have substantially resolved the methodological problems of the instrument, and further studies have demonstrated that the PSPP-R displays adequate psychometric properties [26].

Along with the PSPP, the Perceived Importance Profile (PIP) questionnaire was also developed. The PIP measures the subjective importance assigned to physical conditioning, sport competence, physical strength, and body attractiveness. According to James [27], the perceived importance given to a domain, combined with the subjective evaluation of that domain, affects how situations will shape self-esteem. Therefore, the decision to devote time and energy to a behavior (in our case, physical activity) is assumed to be associated with greater importance in this domain to justify commitment to the current behavior and avoid a perceived inconsistency between attitude and behavior. Subsequent studies have presented evidence supporting James’s individual importance hypothesis [24,26]. These studies also suggested that individuals who assign high importance to a physical domain but who do not perceive themselves as competent in this area are more likely to report lower global self-esteem. Furthermore, findings showed that the revised version of the PIP also presents an acceptable fit to the data [24].

Furthermore, the PSPP-R has been validated across several nationalities (e.g., Sweden, United Kingdom, Turkey, Germany, Greece, Portugal, and Spain) [24,28,29,30,31]. In particular, two studies with older children [30,31] have validated the Spanish version of the PSPP (the Physical Self Questionnaire, PSQ), showing evidence for a four-factor structure. According to the authors, the absence of the physical conditioning factor is due to the way in which primary education is organized. In Spain, physical education in primary school emphasizes content related to image, bodily expression, skills and competencies, and health through play. Meanwhile, aspects related to the physical condition are postponed until secondary education. Although the factorial structure of the PSPP was found to be culturally uniform in several countries, this difference in the Spanish context seems to indicate that contextual variables may affect the inner configuration of the physical self-concept during development.

In Italy, the measurement of physical self-concept has been recognized as a useful instrument for the assessment of psychological balance and mental health. However, only the Physical Self-Description Questionnaire has been validated in two forms: long and short [10,32]. In contrast, the PSPP questionnaire for adolescents and adults has not been validated in the Italian context, not even in its revised form. Despite its relevance for the self-concept and self-esteem, the subjective importance assigned to Physical Self domains has never been included in the assessment.

Based on these considerations, the present study aimed to fill the existing gap in the Italian context through the validation of the revised Physical Self-Perception Profile questionnaire (PSPP-R-IT) and Perceived Importance Profile (PIP-IT). Therefore, we tested the psychometric properties of the PSPP-R-IT and PIP-IT Italian versions.

Given the differences observed in the Spanish cultural context with respect to the uniformity found in other nationalities, on one hand, and the possible similarities between Spanish and Italian cultures, on the other hand, we also considered it necessary to test the factor structure of the PSPP-R in Italian adolescents. Thus, we hypothesized that the Italian versions of the PSPP-R and PIP have a hierarchical structure with a four-factor structure at the subdomain level and the Physical Self-Worth factor at the domain level.

In addition, we assessed the internal consistency, temporal stability, and criterion validity.

## 2. Materials and Methods

### 2.1. Participants

A total of 911 students from two independent samples participated in this study. A probabilistic sampling strategy stratified by gender and age was performed. Once the data were collected, all invalid or incomplete questionnaires were eliminated. In addition, questionnaires that were outside the age range considered for this study were excluded. The final sample consisted of 843 Italian high school students from southern Italy aged 15–20 years old (300 males and 543 females, mean age 17.50 ± 1.02 years). These students came from 15 secondary schools and completed the PSPP-R-IT and PIP-IT questionnaires to examine their factorial validity and structural validity.

A second convenient sample of 68 Italian students aged 16–19 years old (48 males and 20 females, mean age 17.47 ± 1.04 years) was recruited from the same high schools. These students completed the PSPP-R-IT and PIP-IT twice within a one-week period to test their temporal stability.

### 2.2. Measures

Participants responded to questions assessing gender, age, school, grade, city of origin, and educational qualifications.

Two questions evaluated the participants’ engagement in physical activity outside their school’s physical education classes. Students received the definition of physical activity as any body movement that leads to energy expenditure, including organized, structured, and repetitive exercises, as well as sports dance [33]. First, participants were asked whether they had engaged in physical activity outside of their school’s physical education classes during the past six months. Three options were provided: 1 = “No, I have never practiced physical activity or sport outside my school’s physical education class”, 2 = “No, I had to stop my practice because…”, or 3 = “Yes, I usually practice physical activity or sport outside my school’s physical education class”. The students’ responses formed three groups: non-practitioners, dropouts, and practitioners. Second, students who were physically active outside of school were asked to indicate how often they had exercised or played a sport outside of physical education classes in the previous six months. There were seven response options: 0 = 1–3 times per month or less, 1 = 1 day per week, 2 = 2 days per week, 3 = 3 days per week, 4 = 4 days per week, 5 = 5 days per week, and 6 = 6 or more days per week. These responses were used to assess the frequency of students’ participation in physical activities beyond school [4].

The short version of the Physical Self-Description Questionnaire and the Italian Revised Physical Self-Perception Profile were used to measure physical self-concept.

Italian Physical Self-Description Questionnaire (PSDQ-S): The Italian short version of the Physical Self-Description Questionnaire (PSDQ-S) [32]—a modification of the original English edition [34]—was utilized to assess physical self-concept. The PSDQ-S is a self-reported questionnaire that includes 43 items evaluating two broad dimensions—self-esteem and physical self-concept—along with nine subscales related to physical self-concept, namely, health, physical appearance, body fat, physical activity, sports competence, coordination, flexibility, strength, and endurance. Each subscale contains four items, except for the Strength subscale, which comprises three items. Respondents evaluate each declarative statement (e.g., “I am good at most sports”) using a 6-point scale ranging from 1 (“false”) to 6 (“true”). The PSDQ-S effectively reduces the length of the original questionnaire while preserving its psychometric integrity, as evidenced by Cronbach’s Alpha coefficients ranging from 0.77 to 0.91 [4]. In this study, the Cronbach’s Alpha coefficients were found to range from 0.77 to 0.86.

Italian Revised Physical Self-Perception Profile (PSPP-R-IT): The original PSPP-R is a self-reported questionnaire consisting of 42 positively worded item statements. Participants are asked to indicate their responses on a 4-point Likert-type scale ranging from 1 (“Not true at all for me”) to 4 (“Really true for me”) [24].

Similar to the PSPP-R, the Italian adaptation (PSPP-R-IT) contains 30 competence items divided into four subdomains: sports competence (SC), physical conditioning (PC), body attractiveness (BA), and physical strength (PS). Additionally, it incorporates Physical Self-Worth (PSW) with six items at the domain level and General Self-Worth (GSW) as an overarching factor, which also comprises six items, for a total of forty-two items. In this investigation, the Cronbach’s alpha coefficients were found to range from 0.89 to 0.95.

The revised Italian version of the PIP (PIP-IT) consists of 30 items organized into five scales: Physical Conditioning Importance (PCIMP), Sport Competence Importance (SCIMP), Physical Strength Importance (PSIMP), Body Attractiveness Importance (BAIMP), and Physical Self-Worth Importance (PSWIMP). Instead of 2 items per scale, like in the original PIP [1], the revised version includes 6 items per scale (except for the BAIMP scale, which consists of 7 items). Each competence item is matched with a corresponding importance item, resulting in a comprehensive total of 72 items. For instance, a perceived sports competence statement such as “I do very well in all kinds of sports” pairs with its related importance query: “How important is it to you that you do well in all kinds of sports?” The Cronbach’s alpha coefficients observed in this study range from 0.87 to 0.93.

### 2.3. Procedures

This research was carried out in a collective form after ensuring the cooperation of the school principals involved. Before administering the questionnaires, parents of the students were requested to complete a consent form and provided with comprehensive information regarding this study, including its aims and the data collection process. The consent forms were given to a designated teacher, who was responsible for their distribution and collection. Subsequently, a paper-and-pencil questionnaire that included all relevant measures was distributed in classrooms during regular school hours. Each class completed the questionnaires in a single session. Before completing the questionnaire, participants were informed about this study’s objectives and assured of the anonymity and confidentiality of their responses. They were asked for their consent and informed that they had the right to withdraw from participation at any time. All adolescents included in the sample agreed to participate in this study. Participants received instructions on how to fill out the questionnaires, as well as an explanation of physical activity, emphasizing that there were no correct or incorrect answers. Completing these questionnaires took each student approximately 20 min on average.

All studies were conducted in accordance with the ethical principles of the Declaration of Helsinki and approved by the Kore University of Enna Internal Review Board on 13 May 2016.

With regard to the validation of the psychometric properties of the Italian versions of the PSPP-R and PIP, the language-specific versions of the questionnaires were administered to the Italian sample using Brislin’s [35] translation procedure. First, we translated the instrument into Italian. Next, two native English speakers, unaware of the original text, back-translated it into English. After that, we adapted the instruments for cultural relevance and validated them through review testing to ensure accuracy and reliability in cross-cultural contexts. This protocol minimizes cultural biases through identifying and addressing any cultural influences that could alter the intended message. The process is structured and replicable, involving direct translation, back-translation by an independent translator, and a comparison to refine accuracy. Relying on separate translators for each stage ensures independence and objectivity, reducing personal or contextual bias.

The PSPP-R and PIP items [24,26] were translated into Italian by the first author and re-translated by two other native English speakers. The re-translated questionnaires were then compared with the originals, and no changes were applied. The resulting questionnaires were administered to a small sample of students (1 junior high school class and 1 senior high school class), who commented on their understanding of the items. In this preliminary administration, no item comprehension difficulties were found. Therefore, the PSPP-R-IT and PIP-IT were administered to the first sample of participants (*n* = 1801) to examine their factorial, structural, criterion validity, and internal consistency. Subsequently, the second sample of participants (*n* = 68) was administered the PSPP-R-IT and PIP-IT twice over a one-week period, in order to test for temporal stability. As with the other administrations, permission was sought from school principals to administer the instruments. The instruments were administered in the classrooms of the schools. Students participated voluntarily after obtaining parental consent, were guaranteed confidentiality of their responses, and were told that there were no right or wrong answers.

### 2.4. Data Analysis

In the preliminary analysis, the presence of univariate outliers and the normality of the distributions of the scales used were checked in order to choose the most appropriate statistical tests. The skewness, kurtosis, Kolmogorov–Smirnov, and Shapiro–Wilk tests revealed normal distributions.

Descriptive analyses were performed on the sample according to gender, age groups, and the practice of physical activities.

Means, standard deviations, and Cronbach’s alpha values were calculated. A one-way analysis of variance (ANOVA) was conducted for each dependent variable, in order to compare gender and grade differences. Pairwise comparisons among grades were assessed using Tukey’s honestly significant difference (HSD) post hoc tests [36]. Pearson’s r was employed to analyze the correlations between all variables.

A Multivariate Analysis of Variance was performed using physical activity participation groups as a between-subjects factor, gender as a covariate, and the PSPP-R-IT and PIP-IT as dependent variables.

**Figure 1:**
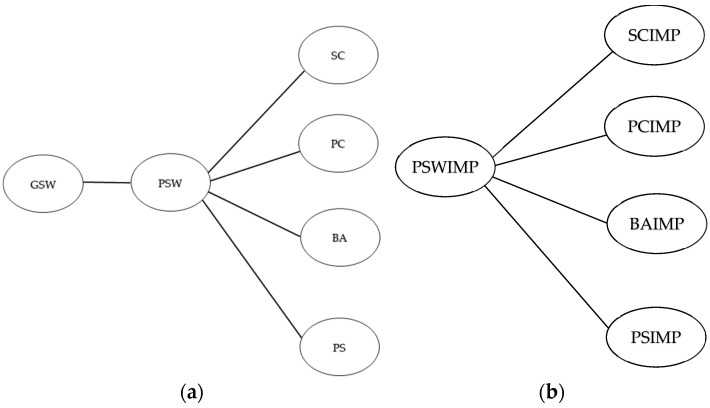
Confirmatory factor analysis models of the PSPP-R and the PIP: (**a**) PSPP-R Model with General Self-Worth as a superordinate factor and Physical Self-Worth at the domain level to explain the covariance between the sub-domains. SC: sports competence; PC: physical conditioning; BA: body attractiveness; PS: physical strength; PSW: Physical Self-Worth; GSW: General Self-Worth. (**b**) PIP model with a higher-order Physical Self-Worth importance factor to explain the covariance between the sub-domains. SCIMP: Sports Competence Importance; PCIMP: Physical Conditioning Importance; BAIMP: Body Attractiveness Importance; PSIMP: Physical Strength Importance; PSWIMP: Physical Self-Worth Importance.

To test the psychometric properties of the Italian versions of the PSPP-R and PIP, descriptive statistics and correlations between variables were calculated. The LISREL program (version 8.8 for Windows) [37] was used to examine the hypothesized six-factor solution of the PSPP-R-IT measurement model using confirmatory factor analysis. The hypothesized hierarchical structure among the PSPP-R subscales proposed by Fox and Corbin [1] (Figure 1a) was examined.

An alternative model was also tested (model B) [38]. In model B, only the relationships between the PSW and the subscales SC, PC, BA, and PS were tested.

The following fit indices were used: (a) χ^2^, (b) the non-normalized fit index (NNFI), (c) the comparative fit index (CFI), and (d) the root mean square error of approximation (RMSEA). The 90% confidence intervals of the RMSEA were also calculated. NNFI and CFI values greater than 0.90 and 0.95, respectively, are considered to reflect an acceptable and excellent fit to the data [39,40]. RMSEA values less than or equal to 0.05 are considered to reflect a close and acceptable fit [41].

Considering that the PSPP-R-IT data in the present study showed multivariate kurtosis (normalized estimate of Mardia = 59.797), the reported values of the fit indices (i.e., χ^2^, NNFI, CFI, RMSEA) were estimated using the robust method [42,43]. Finally, a MANOVA was performed to examine differences in the students’ scores on the PSPP-R-IT subscales by gender, by grade, and by physical activity practice.

In addition, a five-factor solution of the PIP-IT measurement model was examined using the LISREL 8.80 software. The hierarchical structure among the PIP subscales proposed by Fox [2] (Figure 1b) was examined. As the PIP-IT presented the same non-normality problems, the PIP model was also estimated using the robust maximum likelihood estimation method of Satorra and Bentler [43].

## 3. Results

Descriptive statistics and correlations between all variables in the full sample were calculated, and univariate and multivariate normality assumptions were verified. Although the univariate skewness and kurtosis values were within acceptable limits, Mardia’s multivariate kurtosis index indicated the presence of multivariate outliers. Compliance with the multivariate normality assumption ensures that factor analyses adequately test the fit of the model to the data [44]. Therefore, all cases with a Mardia index above the critical value were eliminated, reducing the number of participants from 1809 to 843 subjects for the PSPP-R-IT. Due to the reduction in the sample size, the distribution obtained was not compatible with the multivariate normal distribution. Therefore, a robust maximum likelihood method was used for model estimation in order to avoid contamination of the estimates due to slight violations of the normality assumption [43]. This approach, which incorporates a scaling adjustment for χ^2^ (S-B χ^2^), has been shown to yield the most reliable statistics across different distributions and sample sizes [45]. For the PIP-IT subscales, Mardia’s index also exceeded the critical value, so the number of subjects was reduced to 431 for this procedure and a robust maximum likelihood method was used to estimate the model.

The univariate skewness and kurtosis values and Cronbach’s alpha for the subscales of the PSPP-R-IT and the PIP-IT are provided in Table 1.

Descriptive statistics and correlations between all variables for the reduced sample (*n* = 843) are presented in Table 2.

### 3.1. Structural Validity of the PSPP-R-IT

MODEL A: Confirmatory factor analysis showed an adequate fit of Model A, with the following values obtained for the six-factor solution of the PSPP-R-IT measurement model: S-Bχ^2^ (584, *n* = 843) = 2637.662, *p* < 0.001, NNFI = 0.990, IFI = 0.987, CFI = 0.987, RMSEA = 0.064 (90% CI: 0.062–0.067; *p* < 0.05). The GFI = 0.85, AGFI = 0.83, RMR = 0.036, and sRMR = 0.049 values indicate an adequate overall fit of the model to the data.

For each of the factors, the mean variance extracted was greater than 0.50 and the composite reliability was greater than 0.70. The coefficient of determination (or *R*^2^) values for SC, PC, BA, and PS were all above 0.50 (ranging from 0.556 to 0.798). The factor saturations of the individual items are shown in Table 3.

The factor saturations of the SC, PC, BA, and PS subdomains on PSW were 0.68, 0.77, 0.64, and 0.83, respectively, all with *p* < 0.05. In addition, PSW saturation on GSW was 0.65 (*p* < 0.05).

An alternative structural model was also tested.

MODEL B: Model B also fit the data: S-Bχ^2^ (575, *n* = 843) = 1037.329, *p* < 0.001, NNFI = 0.979, IFI = 0.982, CFI = 0.982, RMSEA = 0.05 (90% CI: 0.046–0.053; *p* = 0.592). The CFI and NNFI indices exceeded a value of 0.90, indicating an overall adequate fit of the model to the data. In addition, the RMR = 0.053, sRMR = 0.058, GFI = 0.91, and AGFI = 0.893 indices demonstrated a very good fit of the model to the data. However, the average variance extracted was less than 0.50 for the PC and PSW factors, while the composite reliability was greater than 0.70 for each of the five factors. The *R*^2^ values for the SC, PC, BA, and PS subscales were all greater than 0.50 (ranging from 0.536 to 0.948). The factor saturations of the SC, PC, BA, and PS subdomains on PSW were 0.63, 0.95, 0.89, and 0.52, respectively (all significant; *p* < 0.05). However, the fits of models C and D to the data were less adequate.

### 3.2. Structural Validity of the PIP-IT

Confirmatory factor analysis showed a good fit of the model to the data, with the following values obtained for the five-factor solution of the model: S-Bχ^2^ (395, *n* = 431) = 1384.529, *p* < 0.001, NNFI = 0.982, IFI = 0.983, CFI = 0.983, RMSEA = 0.076 (90% CI: 0.072–0.080; *p* < 0.001). The GFI = 0.82, AGFI = 0.79, RMR = 0.02, and sRMR = 0.047 values indicate an overall good fit of the model to the data. In each of the factors, the mean of the average variance extracted was greater than −50, while the composite reliability was greater than −70. The *R*^2^ values for the SCIMP, PCIMP, and BAIMP subscales were all above 0.50 (0.936, 0.903, and 0.912, respectively), with the exception of PSIMP (0.335). The factor saturations of the individual items are shown in Table 4. The factor saturations of the SCIMP, PCIMP, BAIMP, and PSIMP subdomains in the PSWIMP were 0.653, 0.683, 0.870, and 0.727, respectively, all with *p* < 0.05.

### 3.3. Internal Consistency and Temporal Stability

The Cronbach’s alpha coefficients for all subscales of the PSPP-R-IT were satisfactory, ranging between 0.866 and 0.947 (Table 1). To examine the temporal stability of the PSPP-R-IT, 68 students (sample 2) completed the questionnaire twice in one week.

The intraclass correlation coefficients were satisfactory for all subscales of the PSPP-R-IT (SC: *r* = 0.856, *p* < 0.001; PC: *r* = 0.853, *p* < 0.001; BA: *r* = 0.844, *p* < 0.001; PS: *r* = 0.748, *p* < 0.001; PSW: *r* = 0.813, *p* < 0.001), with the exception of the GSW scale, which showed a lower correlation (*r* = 0.585, *p* < 0.001) (Table 5).

The intraclass correlation coefficients were satisfactory for all subscales of the PIP-IT (SCIMP: *r* = 0.774, *p* < 0.001; PCIMP: *r* = 0.714, *p* < 0.001, BAIMP: *r* = 0.797, *p* < 0.001, PSIMP: *r* = 0.690, *p* < 0.001, PSWIMP: *r* = 0.697, *p* < 0.001 (Table 6).

### 3.4. Criterion Validity

The correlations between the subscales of the PSPP-R-IT, participation in physical activities, and hours spent on sedentary activities are presented in Table 5. The correlations with the frequency of sports practice were all positive and significant, with the exception of the GSW scale.

There were no significant correlations between the PSPP-R-IT subscales and hours spent on sedentary activities (TV, PC, and consoles; Table 7).

The correlations between the subscales of the PSPP-R-IT and the subscales of the PSDQ-S are presented in Table 2. All correlations were in the expected direction and were statistically significant. In particular, the SC and PC subscales of the PSPP-R-IT correlated positively with the SC subscale of the PSDQ-S (*r* = 0.797 and 0.735, respectively), and the physical appearance and PS subscales of the PSPP-R-IT correlated positively with the corresponding subscales of PSDQ-S (*r* = 0.644 and 0.777, respectively). In addition, BA and PSW in the PSPP-R-IT correlated positively with the Physical Self-Esteem subscale of the PSDQ-S (*r* = 0.734 and 0.799, respectively).

A one-way MANOVA, with sports participation (participants versus non-participants) as an independent variable and students’ scores on the PSPP-R-IT subscales as dependent variables, showed a significant multivariate effect (F(6, 1802) = 34.61, *p* < 0.001, partial η^2^ = 0.31, Wilks’ λ = 0.691).

ANOVAs revealed that students who practiced physical activities compared to those who did not scored significantly higher on all PSPP-R-IT subscales (SC: F(1, 1807) = 591.166, *p* < 0.001, partial η^2^ = 0.247; PC: F(1, 1807) = 610.253, *p* < 0.001, partial η^2^ = 0.252; BA: F(1, 1807) = 52.944, *p* < 0.001, partial η^2^ = 0.028; PS: F(1, 1807) = 347.512, *p* < 0.001, partial η^2^ = 0.161; PSW: F(1, 1807) = 83.768, *p* < 0.001, partial η^2^ = 0.044; GSW: F(1, 1807) = 23.936, *p* < 0.001, partial η^2^ = 0.013).

A one-way MANOVA, with sports participation as an independent variable and students’ scores on the PIP-IT subscales as dependent variables, showed a significant multivariate effect (F(5, 1801) = 113.715, *p* < 0.001, partial η^2^ = 0.24, Wilks’ λ = 0.760).

ANOVAs revealed that students who practiced physical activities, compared to those who did not, also scored significantly higher on all PIP-IT subscales (SCIMP: F(1, 1805) = 410.002, *p* < 0.001, partial η^2^ = 0.185; PCIMP: F(1, 1805) = 439.402, *p* < 0.001, partial η^2^ = 0.196; BAIMP: F(1, 1805) = 4.575, *p* = 0.03, partial η^2^ = 0.003; PSIMP: F(1, 1805) = 254.278, *p* < 0.001, partial η^2^ = 0.123; PSWIMP: F(1, 1805) = 19.041, *p* < 0.001, partial η^2^ = 0.010).

### 3.5. Differences by Gender and Grade

An analysis of variance was performed to examine differences by gender and grade separately. Comparisons by gender showed that males had significantly higher means than females on all subscales of the PSPP-R-IT and on the SCIMP, PCIMP, and PSIMP subscales of the PIP-IT. Females had significantly higher means on the PSWIMP subscale. No significant differences were observed for BAIMP.

As for the grades, there was a difference between the fourth and fifth (last) grades of high school. In particular, in the fifth grade, the means of all the PSPP-R-IT subscales are significantly lower than in the two previous grades, with the exception of BA. Comparisons via Tukey’s HSD analysis showed that, on these subscales, the data formed two groups—one including third- and fourth-graders and the other including fifth-graders. In the PIP-IT subscales, on the other hand, there were no statistically significant differences between grades.

## 4. Discussion

A valid and reliable instrument that measures physical self-concept and its subjective importance can improve our understanding of the role that this cognitive process plays in the development and education of adolescents. As has been previously stated, in adolescent years, when psychosocial processes acquire an important value for well-being and mental health, having a good approach to self-concept is important. Although another validated instrument exists in Italy to assess physical self-concept—namely, the PSDQ [10,32]—there currently exists a lack of instruments to evaluate both the individually perceived Physical Self domains and the importance assigned to various dimensions of one’s Physical Self, such as experiences related to sports competence, fitness, and strength. Perceived importance refers to the subjective value that each person assigns to the different dimensions of the physical self-concept. The theoretical assumption of the PSPP-R and PIP is that the combination of competence and importance that people attribute to a specific area of evaluation will affect their self-esteem. Only the areas to which people assign the most value will contribute to overall feelings of self-esteem [27]. The importance assigned to the physical self-concept allows us to understand the hierarchy in which adolescents place the different dimensions of perceived physical appearance and sports experience [4].

Therefore, this study aimed to examine the psychometric properties of the Italian version of the PSPP-R with the PIP-IT in order to provide, for the Italian context, a comprehensive instrument for understanding the development of the Physical Self and global self-esteem dimensions. The factor structure, internal consistency, temporal stability, and criterion validity of the PSPP-R-IT and the PIP-IT were examined.

The obtained results confirmed the six-factor structure of the PSPP-R-IT and the five-factor structure of the PIP-IT, as well as the hypothesized hierarchical structures among the subscales of these questionnaires [26]. The results of the confirmatory factor analyses showed a clear six-factor solution for the PSPP-R-IT (model A) (Figure S1). The five-factor structure also presented an adequate fit of the model to the data (model B); however, the fit indices were better for the six-factor model. Furthermore, the results of the six-factor model supported the hypothesized hierarchical structure of the PSPP-R-IT. In particular, the four subdomains of the PSPP-R-IT (SC, PC, BA, and PS) are hierarchically related to the more global perceptions of PSW, located at the domain level, and GSW was located at the apex of this hierarchical model.

Regarding the PIP-IT, the hierarchical structure was confirmed, with the four subdomains (SCIMP, PCIMP, BAIMP, and PSIMP) being hierarchically related to PSWIMP (Figure S2).

Therefore, the present results are consistent with theoretical and empirical evidence regarding the multi-dimensional and hierarchical structure of physical self-concept [24,26].

The PSPP-R-IT and PIP-IT had satisfactory internal consistency and remained stable over a one-week period. With regard to the criterion validity of the PSPP-R-IT and PIP-IT, the subscales of the PSPP-R-IT correlated significantly with the corresponding subscales of the PSDQ-S. These results provided evidence supporting the criterion validity of the PSPP-R-IT, while the criterion validity of the PIP-IT can be partially accepted. The PIP-IT subscales correlated significantly with the corresponding subscales of the PSDQ-S with the exception of BAIMP, which did not correlate significantly with either the body fat or health subscales.

Males and females reported significantly different levels on all scales of the PSPP-R-IT. In particular, males scored higher on the physical self-concept subdomains, which aligns with previous research [23,45] but contrasts with the findings of Kolovenis et al. (2013). Regarding the PIP-IT, males reported significantly higher levels in SCIMP, PCIMP, and PSIMP than females. In contrast, females had higher means in the PSWIMP. No significant differences were found in BAIMP. The physical self-concept of students declines with age, as has been well documented in previous studies [2,4,16,47,48,49]. In our study, we observed a significant decrease in the means between the fourth and fifth grades in the SC, PC, PS, and PSW subdomains, but not in BA. Additionally, SCIMP, PCIMP, and PSIMP decreased with age, whereas BAIMP increased with age. Our results suggest that the age of 17 years is a critical point after which participation in physical activity significantly tends to decline. Taken together, all these results are consistent with previous findings [24,26,29], supporting the validity of the PSPP-R-IT and PIP-IT criteria. Moreover, the Italian version of the PSPP-R showed adequate psychometric properties and can therefore be used in research involving Italian adolescents.

As has been widely demonstrated in the literature, physical self-concept becomes particularly important during adolescence, mainly because of the need to adjust to physical changes and to re-establish a sociopsychological balance with one’s new self-image [4]. The perceived cognitive dissonance between high levels of domain importance and low levels of competence leads to lower levels of overall self-esteem. In our study, as suggested in previous research [26], body attractiveness competence and importance differ from other subdomains of the Physical Self (e.g., no significant differences between genders and practice in the importance of body attractiveness). In fact, studies supporting James’s individual importance hypothesis [24,26] have provided evidence for the domains of sports and strength, which are less related to global self-esteem. In contrast, the more that subdomains explain the variance in global self-esteem and Physical Self-Worth, the less support is provided for James’s importance hypothesis, as was the case for the body attractiveness subdomain. Indeed, body attractiveness showed the strongest relationship with global self-esteem, but no evidence was found regarding the moderating effect of the importance assigned to physical attractiveness in the relationship between global self-esteem and the body. Thus, the body subdomain seems to work differently than the others [24,26].

In our study, the body attractiveness subscale seemed to be perceived as a dimension separate from the other subscales. This evidence is consistent with previous research on self-concept, which demonstrated that the physical aspect is a distinct component with respect to physical ability [50]. The results revealed negative estimates between the importance of body attractiveness and the importance of physical conditioning in the PIP Model (Figure S2). The results seem to indicate that, in our sample, the importance of being physically attractive is perceived as different (and opposite) to the importance of being in good physical condition. For instance, an athlete might believe it more important that she or he is in excellent physical condition but not important that a particular part of her or his body is unattractive because of the physical activities she or he engages in. Conversely, a sedentary student might perceive that it is more important to perceive his or her body as attractive and less important to be fit. However, this aspect does not disregard the hierarchy that each adolescent establishes among the various dimensions of the physical self-concept in relation to his or her bodily experience. The distinction between physical conditioning and body attractiveness affirms the existence of independent dimensions within the physical self-concept construct, as postulated in accordance with the theoretical framework [4].

## 5. Conclusions

This study aimed to assess the psychometric properties of the Italian versions of the PSPP-R and the PIP.

The key findings of our study can be summarized as follows:

- Confirmatory factor analyses revealed a clear six-factor solution for the PSPP-R-IT, presenting better fit indices than the five-factor solution. The six-factor model has a hierarchical structure in which General Self-Worth is at the apex, Physical Self-Worth is at the domain level, and four factors are at the subdomain level (sport competence, physical conditioning, physical strength, and body attractiveness).
- The hierarchical structure of the PIP-IT was confirmed, with the four importance subdomains being hierarchically related to Physical Self-Worth Importance.
- Evidence supporting the internal consistency, temporal stability, and criterion validity of the questionnaires was presented.
- Males had significantly higher means than females on all subscales of the PSPP-R-IT and on the importance subscales of the PIP-IT, with the exception of the Body Attractiveness Importance subscale. Females had significantly higher means in the Physical Self-Worth Importance domain.
- There was a difference between the fourth and fifth (last) years of high school, where the means of all the PSPP-R-IT subscales were significantly lower than in the two lower years, with the exception of body attractiveness.
- Students who practice physical activities, compared to those who do not, scored significantly higher on all PSPP-R-IT and PIP subscales.
- There were no significant correlations between the physical self-concept and time spent in sedentary activities.

This study has some limitations. The first limitation is that cross-sectional research designs do not enable an analysis of the developmental processes of physical self-concept in adolescents using a longitudinal approach.

Second, although the sampling was stratified by gender and age, the participants involved were from a homogeneous geographical area in southern Italy.

Third, participation in physical activity was not assessed using a validated self-reported evaluation questionnaire; rather, it was measured through two items that determine the type of participation and its frequency. This approach did not allow for structured scores related to daily physical activity to be obtained.

Despite some limitations, this study validated a useful instrument which allows for a more comprehensive understanding of the interplay between competence and the importance of physical domains. Therefore, the Italian versions of the PSPP-R and PIP can be effectively used in research with Italian adolescents and adults.

## Figures and Tables

**Table 1:** Cronbach’s alpha, skewness, and univariate kurtosis of the subscales of the Physical Self-Perception Profile—Revised (PSPP-R).

	Range	α	A	C
PSPP-R-IT (*n* = 843)				
Sports Competence	1–4	0.941	0.269	−0.913
Physical Conditioning	1–4	0.931	0.167	−0.946
Body Attractiveness	1–4	0.921	−0.063	−0.724
Physical Strength	1–4	0.947	0.431	−0.706
Physical Self-Worth	1–4	0.927	−0.321	−0.482
General Self-Worth	1–4	0.888	−0.556	0.318
PIP-IT (*n* = 431)				
Sports Competence Imp.	1–4	0.931	0.034	−0.502
Phys. Conditioning Imp.	1–4	0.896	−0.218	−0.075
Body Attractiveness Imp.	1–4	0.866	−0.333	0.563
Phys. Strength Imp.	1–4	0.931	0.121	−0.369
Phys. Self-Worth Imp.	1–4	0.886	−0.509	1.530

Legend: α: Cronbach’s alpha; A: skewness; C: kurtosis. Adapted from Nicolosi [46].

**Table 2:** Means, standard deviations, and correlations among all variables.

	M	DS	1	2	3	4	5	6	7	8	9	10	11	12	13	14	15	16	17	18	19	20	21	22
1. Sports Competence (PSPP-R-IT)	2.212	0.773	—																					
2. Physical Conditioning (PSPP-R-IT)	2.305	0.781	0.918 **	—																				
3. Body Attractiveness (PSPP-R-IT)	2.393	0.746	0.683 **	0.776 **	—																			
4. Physical Strength (PSPP-R-IT)	2.159	0.765	0.831 **	0.836 **	0.625 **	—																		
5. Physical Self-Worth (PSPP-R-IT)	2.728	0.712	0.696 **	0.788 **	0.882 **	0.643 **	—																	
6. General Self-Worth (PSPP-R-IT)	2.971	0.637	0.531 **	0.576 **	0.648 **	0.465 **	0.740 **	—																
7. Sports Competence Imp. (PIP-IT)	2.343	0.696	0.748 **	0.698 **	0.454 **	0.660 **	0.442 **	0.301 **	—															
8. Physical Conditioning Imp. (PIP-IT)	2.591	0.637	0.682 **	0.672 **	0.441 **	0.613 **	0.427 **	0.285 **	0.852 **	—														
9. Body Attractiveness Imp. (PIP-IT)	2.866	0.546	0.191 **	0.209 **	0.271 **	0.173 **	0.171 **	0.122 **	0.409 **	0.478 **	—													
10. Physical Strenght Imp. (PIP-IT)	2.256	0.675	0.633 **	0.619 **	0.411 **	0.685 **	0.389 **	0.240 **	0.819 **	0.805 **	0.420 **	—												
11. Physical Self Worth Imp. (PIP-IT)	3.160	0.507	0.215 **	0.235 **	0.253 **	0.189 **	0.250 **	0.236 **	0.375 **	0.502 **	0.699 **	0.351 **	—											
12. Sports Competence (PSDQ-S)	3.564	1.343	0.847 **	0.816 **	0.565 **	0.713 **	0.596 **	0.405 **	0.672 **	0.629 **	0.136 **	0.541 **	0.151 **	—										
13. Physical Appearance (PSDQ-S)	3.965	1.104	0.496 **	0.537 **	0.693 **	0.447 **	0.632 **	0.547 **	0.303 **	0.290 **	0.245 **	0.273 **	0.187 **	0.461 **	—									
14. Strenght (PSDQ-S)	3.354	1.300	0.721 **	0.740 **	0.544 **	0.826 **	0.565 **	0.394 **	0.574 **	0.535 **	0.141 **	0.588 **	0.113 **	0.731 **	0.492 **	—								
15. Flexibility (PSDQ-S)	3.537	1.285	0.525 **	0.566 **	0.447 **	0.439 **	0.437 **	0.312 **	0.396 **	0.398 **	0.162 **	0.318 **	0.153 **	0.581 **	0.364 **	0.477 **	—							
16. Condition/Endurance (PSDQ-S)	3.221	1.409	0.713 **	0.740 **	0.570 **	0.623 **	0.575 **	0.393 **	0.567 **	0.547 **	0.134 **	0.499 **	0.170 **	0.696 **	0.382 **	0.620 **	0.507 **	—						
17. Coordination (PSDQ-S)	3.986	1.096	0.642 **	0.655 **	0.500 **	0.534 **	0.521 **	0.410 **	0.505 **	0.491 **	0.176 **	0.383 **	0.207 **	0.721 **	0.414 **	0.551 **	0.686 **	0.551 **	—					
18. Physical Activity (PSDQ-S)	2.850	1.577	0.674 **	0.672 **	0.408 **	0.610 **	0.441 **	0.291 **	0.543 **	0.561 **	0.151 **	0.491 **	0.173 **	0.687 **	0.315 **	0.577 **	0.450 **	0.608 **	0.500 **	—				
19. Body Fat (PSDQ-S)	4.755	1.348	0.363 **	0.470 **	0.654 **	0.248 **	0.589 **	0.360 **	0.180 **	0.190 **	0.046	0.112 **	0.098 **	0.313 **	0.417 **	0.193 **	0.337 **	0.382 **	0.359 **	0.165 **	—			
20. Health (PSDQ-S)	5.163	0.943	0.248 **	0.275 **	0.232 **	0.275 **	0.243 **	0.214 **	0.152 **	0.138 **	0.031	0.111 **	0.071 *	0.186 **	0.139 **	0.196 **	0.083 *	0.186 **	0.170 **	0.134 **	0.190 **	—		
21. Physical Self (PSDQ-S)	4.057	1.369	0.610 **	0.703 **	0.812 **	0.539 **	0.853 **	0.647 **	0.372 **	0.360 **	0.090 **	0.315 **	0.136 **	0.580 **	0.652 **	0.554 **	0.456 **	0.542 **	0.534 **	0.398 **	0.637 **	0.218 **	—	
22. General Self-Esteem (PSDQ-S)	4.426	0.884	0.536 **	0.569 **	0.580 **	0.493 **	0.638 **	0.664 **	0.331 **	0.319 **	0.149 **	0.294 **	0.174 **	0.495 **	0.575 **	0.487 **	0.396 **	0.437 **	0.504 **	0.329 **	0.342 **	0.247 **	0.629 **	—
23. Participation in Sports	1.492	0.872	0.508 **	0.489 **	0.271 **	0.436 **	0.309 **	0.185 **	0.409 **	0.402 **	0.041	0.354 **	0.102 **	0.527 **	0.202 **	0.386 **	0.317 **	0.407 **	0.367 **	0.627 **	0.133 **	0.112 **	0.252 **	0.206 **

* *p* < 0.05; ** *p* < 0.01. M: mean; SD: standard deviation; Imp.: importance. Adapted from Nicolosi [46].

**Table 3:** Factor saturations of the individual items of Model A (PSPP-R).

	SC	PC	BA	PS	PSW	GSW
item 1	0.820					
item 7	0.809					
item 13	0.828					
item 19	0.828					
item 25	0.834					
item 31	0.852					
item 2		0.745				
item 8		0.761				
item 14		0.861				
item 20		0.765				
item 26		0.885				
item 32		0.813				
item 3			0.857			
item 9			0.865			
item 15			0.830			
item 21			0.881			
item 27			0.887			
item 33			0.879			
item 4				0.774		
item 10				0.816		
item 16				0.818		
item 22				0.883		
item 28				0.743		
item 34				0.888		
item 5					0.796	
item 11					0.843	
item 17					0.847	
item 23					0.878	
item 29					0.856	
item 35					0.868	
item 6						0.780
item 12						0.685
item 18						0.719
item 24						0.776
item 30						0.866
item 36						0.697

Italian Physical Self-Perception Profile—Revised (PSPP-R-IT): SC: sports competence; PC: physical conditioning; BA: body attractiveness; PS: physical strength; PSW: Physical Self-Worth; GSW: General Self-Worth.

**Table 4:** Factor saturations of the individual items of the PIP.

	SCIMP	PCIMP	BAIMP	PSIMP	PSWIMP
item 1	0.755				
item 6	0.895				
item 11	0.847				
item 16	0.908				
item 21	0.869				
item 26	0.912				
item 2		0.730			
item 7		0.804			
item 12		0.841			
item 17		0.818			
item 22		0.795			
item 27		0.838			
item 3			0.777		
item 8			0.747		
item 13			0.793		
item 18			0.781		
item 28			0.845		
item 31			0.734		
item 4				0.868	
item 9				0.898	
item 14				0.854	
item 19				0.833	
item 24				0.893	
item 29				0.907	
item 5					0.827
item 10					0.774
item 15					0.812
item 20					0.820
item 25					0.781
item 30					0.912

Italian Perceived Importance Profile (PIP-IT): SCIMP: Importance of Sports Competence; PCIMP: Importance of Physical Conditioning; BAIMP: Importance of Body Attractiveness; PSIMP: Importance of Physical Strength; PSWIMP: Importance of Physical Self-Worth.

**Table 5:** Correlations between PSPP-R scales administered one week apart to participants in sample 2.

PSPP-R-IT	SC2	PC2	BA2	PS2	PSW2	GSW2
Sports Competence 1	0.856 **	0.796 **	0.582 **	0.621 **	0.592 **	0.405 **
Physical Conditioning 1	0.828 **	0.853 **	0.626 **	0.611 **	0.676 **	0.486 **
Body Attractiveness 1	0.624 **	0.710 **	0.844 **	0.416 **	0.822 **	0.634 **
Physical Strength 1	0.537 **	0.548 **	0.343 **	0.748 **	0.389 **	0.300 *
Physical Self-Worth 1	0.620 **	0.647 **	0.742 **	0.330 **	0.813 **	0.588 **
General Self-Worth 1	0.321 **	0.334 **	0.506 **	0.162	0.509 **	0.585 **

SC: sports competence; PC: physical conditioning; BA: body attractiveness; PS: physical strength; PSW: Physical Self-Worth; GSW: General Self-Worth; 1: time 1; 2: time 2; * *p* < 0.05; ** *p* < 0.01. Adapted from Nicolosi [46].

**Table 6:** Correlations between PIP scales, administered one week apart, to participants in sample 2.

Perceived Importance Profile	SCIMP 2	PCIMP 2	BAIMP 2	PSIMP 2	PSWIMP 2
Sports Competence Importance 1	0.774 **	0.643 **	0.335 **	0.591 **	0.396 **
Physical Conditioning Importance 1	0.711 **	0.714 **	0.339 **	0.507 **	0.446 **
Body Attractiveness Importance 1	0.367 **	0.408 **	0.797 **	0.340 **	0.768 **
Physical Strength Importance 1	0.567 **	0.489 **	0.302 *	0.690 **	0.270 *
Physical Self-Worth Importance 1	0.296 *	0.348 **	0.476 **	0.162	0.697 **

SCIMP: Sport Competence Importance; PCIMP: Physical Conditioning Importance; BAIMP: Body Attractiveness Importance; PSIMP: Physical Strength Importance; PSWIMP: Physical Self-Worth Importance; 1: Time 1; 2: Time 2; * *p* < 0.05; ** *p* < 0.01. Adapted from Nicolosi [46].

**Table 7:** Correlations between PSPP-R and PIP subscales, frequency of physical activity, and hours of sedentary activity.

	1	2	3	4	5	6	7	8	9	10	11	12
1. Sport Competence	-											
2. Physical Conditioning	0.852 **	-										
3. Body Attractiveness	0.548 **	0.637 **	-									
4. Physical Strength	0.696 **	0.646 **	0.405 **	-								
5. Physical Self-Worth	0.560 **	0.684 **	0.824 **	0.394 **	-							
6. General Self-Worth	0.370 **	0.411 **	0.495 **	0.260 **	0.673 **	-						
7. Sport Competence Imp.	0.699 **	0.676 **	0.338 **	0.547 **	0.337 **	0.238 **	-					
8. Phys. Cond. Imp.	0.578 **	0.639 **	0.276 **	0.431 **	0.267 **	0.166	0.787 **	-				
9. Body Attract. Imp.	0.265 **	0.254 **	0.267 **	0.208 *	0.150	0.053	0.449 **	0.558 **	-			
10. Phys. Strength Imp.	0.538 **	0.506 **	0.269 **	0.592 **	0.236 **	0.094	0.732 **	0.681 **	0.556 **	-		
11. Phys. Self-Worth Imp.	0.174 *	0.206 *	0.225 **	0.150	0.180 *	0.210 *	0.340 **	0.537 **	0.682 **	0.394 **	-	
12. Frequency Exercise	0.400 **	0.493 **	0.247 **	0.386 **	0.228 **	0.152	0.375 **	0.455 **	0.104	0.351 **	0.125	-
13. Time sed. activities	0.106	0.080	0.040	0.130	0.030	−0.010	0.005	−0.030	−0.010	0.015	−0.060	−0.050

* *p*< 0.05; ** *p*< 0.01. Adapted from Nicolosi [46].

## Data Availability

The data presented in this study are available on request from the corresponding author due to privacy.

## Supplementary Materials

The following supporting information can be downloaded at https://www.mdpi.com/article/10.3390/bs14121229/s1: Figure S1. Factor structure of the Italian Physical Self-Perception—Revised (PSPP-R-IT); Figure S2. Factor structure of the Italian Perceived Importance Profile (PIP-IT).
