# Supplementary material for: Psychometric Validation of the Revised Physical Self-Perception Profile: An Italian Context Study
Source: Behav Sci (Basel). 2024 Dec 20;14(12):1229. doi: 10.3390/bs14121229 (PMC11673779; doi:10.3390/bs14121229)
Supplement: Supplementary file 1 [file behavsci-14-01229-s001.zip › Figure S1_1.pdf]

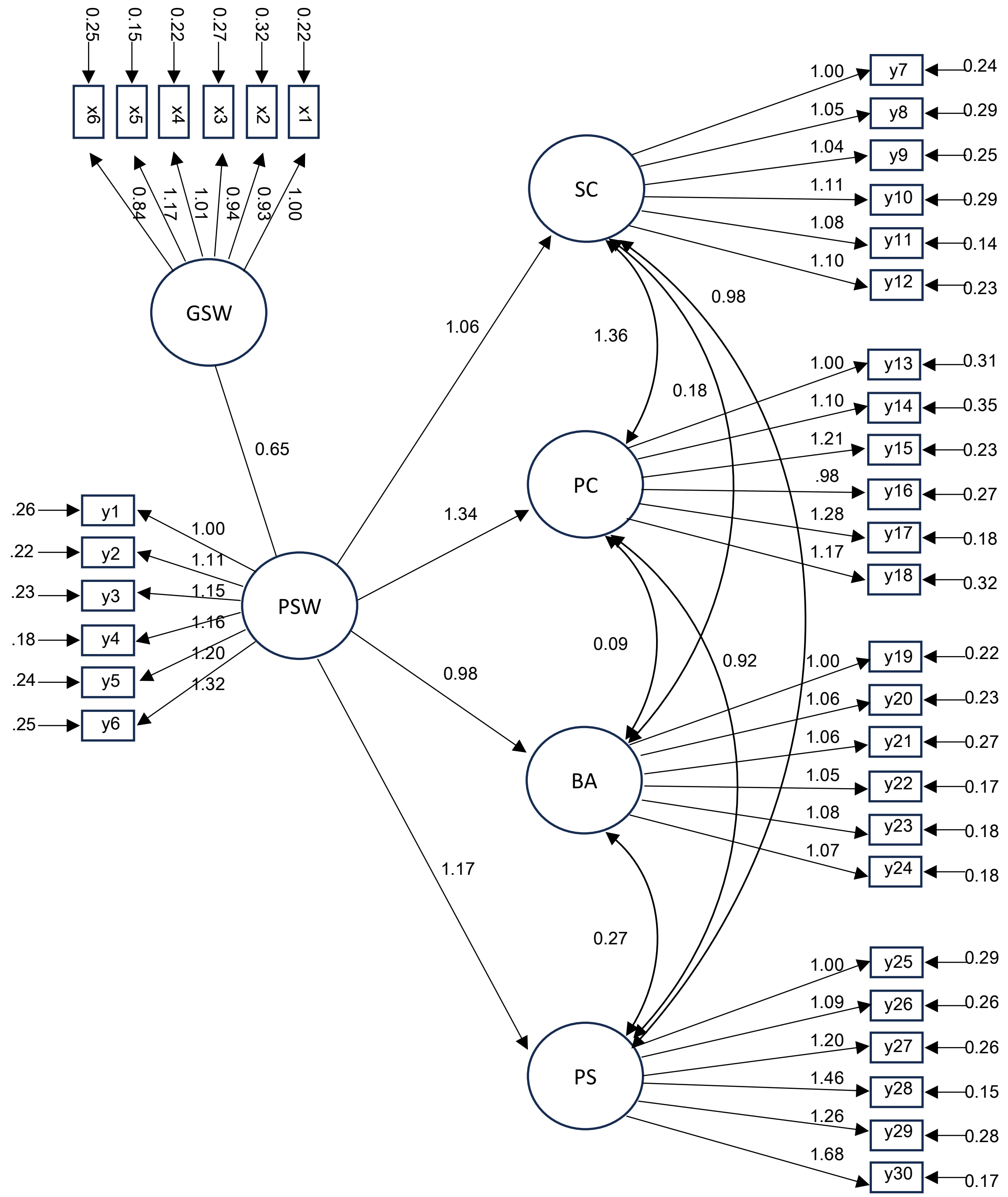

Model A: S-Bχ^2 (584, n = 843) = 2637.662, p < .001, NNFI = .990, CFI = .987, RMSEA = .064 (90% CI: .062–.067; p < .05)

Figure S1. Factor Structure of the Italian Physical Self-Perception – Revised (PSPP-R-IT) GSW: General Self-Worth.SC; PSW: Physical Self-Worth; Sports Competence; PC: Physical Conditioning; BA: Body Attractiveness; PS: Physical Strength.
