# Supplementary material for: Psychometric Validation of the Revised Physical Self-Perception Profile: An Italian Context Study
Source: Behav Sci (Basel). 2024 Dec 20;14(12):1229. doi: 10.3390/bs14121229 (PMC11673779; doi:10.3390/bs14121229)
Supplement: Supplementary file 1 [file behavsci-14-01229-s001.zip › Figure S2_2.pdf]

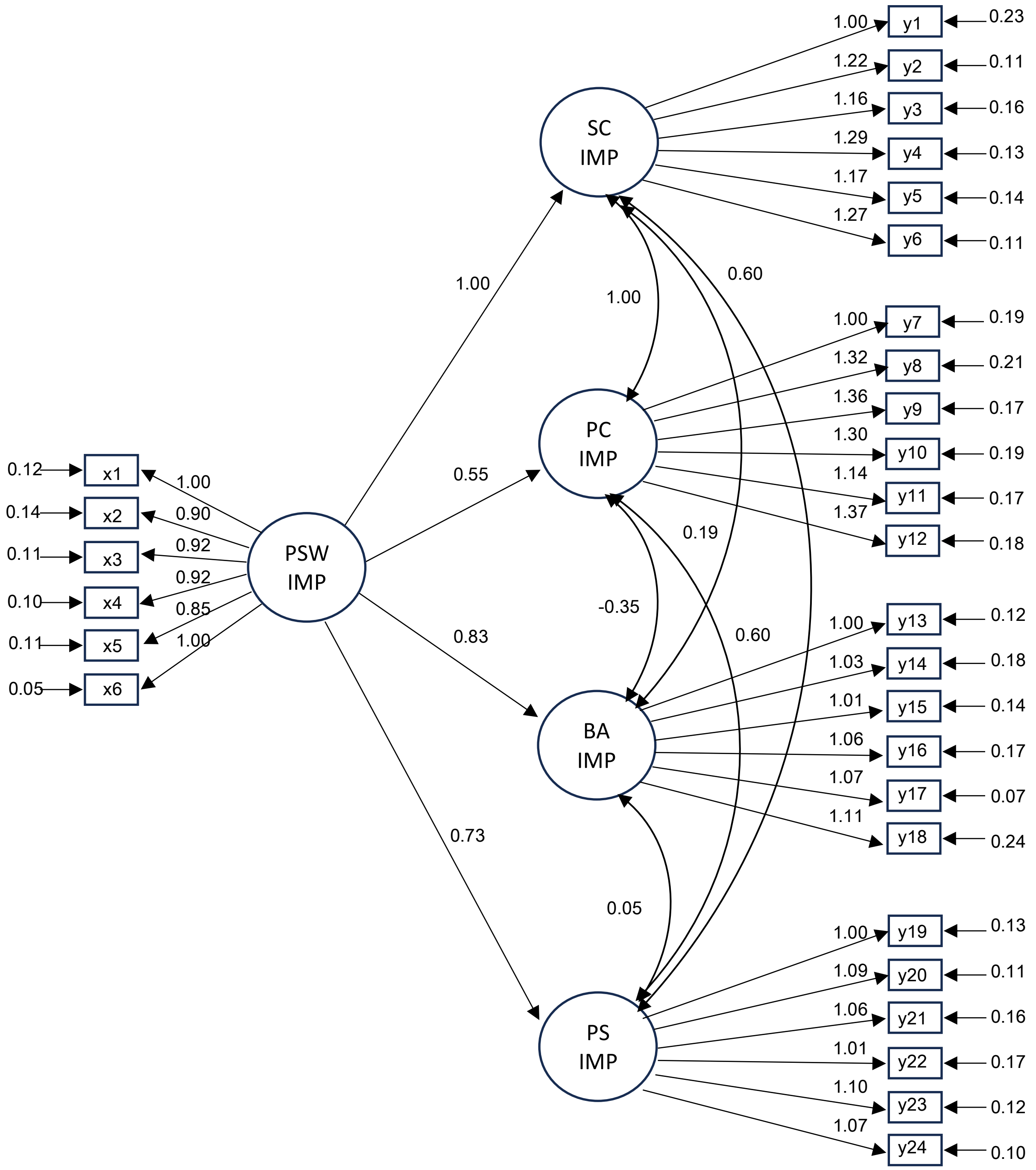

S-Bχ^2 (385, n = 431) = 1384.529, p < .001, NNFI = .982, CFI = .983
RMSEA = .076 (90% CI: .072–.080; p < .001)

Figure S2. Factor Structure of the Italian Perceived Importance Profile (PIP-IT)
PSWIMP: Importance of Physical Self-Worth; SCIMP: Importance of Sports Competence; PCIMP: Importance of Physical Conditioning; BAIMP: Importance of Body Attractiveness; PSIMP: Importance of Physical Strength.
